# Detection of sebaceous gland hyperplasia with dermoscopy and reflectance confocal microscopy

**DOI:** 10.3389/fmed.2023.1194748

**Published:** 2023-06-30

**Authors:** Er-Yi Lin, Lang Rao, Wen-Ju Wang, Yong-Feng Chen

**Affiliations:** ^1^Dermatology Hospital, Southern Medical University, Guangzhou, Guangdong, China; ^2^Department of Dermatology, Chengdu Second People's Hospital, Chengdu, Sichuan, China

**Keywords:** dermoscopy, reflectance confocal microscopy, sebaceous gland, sebaceous gland hyperplasia, non-invasive

## Abstract

**Background:**

Sebaceous gland hyperplasia (SGH) is a benign cutaneous proliferation of the sebaceous glands that are mostly present on the face or the neck of older adults. They typically appear as single or multiple soft umbilicated papules; however, in challenging cases, it can be difficult to distinguish them from trichoepitheliomas, base cell carcinomas, or other tumors. Although pathological results have diagnostic value, the significance of non-invasive examinations in diagnosis and differential diagnosis is also worth exploring.

**Objectives:**

This study aimed to describe the dermoscopic and reflectance confocal microscopy (RCM) features of SGH.

**Methods:**

A total of 31 patients diagnosed with SGH, according to clinical and histopathological standards, were examined using dermoscopy and RCM between March 2018 and January 2022.

**Results:**

Dermoscopically, lesions revealed a yellowish-red background and a faint-yellow background in 25 (80.65%) and six cases (19.35%), respectively. White-yellowish lobulated structures in the center of the lesion were present in 31 patients (100%) and umbilications in 19 patients (61.29%). Crown vessels at the periphery of the lesions were observed in 11 patients (35.48%), whereas irregular linear vessels were observed on the surface of the lesions in 18 patients (58.06%). Under RCM, all lesions presented a honeycomb pattern in the epidermis and the typical morulae-shaped sebaceous lobules in the dermis. A dilated follicular infundibulum was observed in 15 patients (48.39%) and dilated vessels in 26 patients (83.87%).

**Conclusion:**

Dermoscopy and RCM enabled us to describe the imaging features of SGH. Combining these two useful tools provides a non-invasive basis for accurate clinical diagnosis.

## Introduction

Sebaceous gland hyperplasia (SGH) is a benign cutaneous proliferation of the sebaceous glands that primarily affects the face and increases with ultraviolet-B (UVB) exposure and aging. It has been reported to occur in ~1% of the healthy population, mainly men or boys (1, 2). The typical manifestations of SGH are skin-colored or whitish-yellow, normally umbilical papules that vary in size from 2 to 9 mm (3). Diagnosis is usually straightforward, based on clinical and dermoscopic findings; however, histopathology is the gold standard in more complex cases where malignancy cannot be ruled out. However, histopathology is an invasive examination that is not easily accepted by patients, especially for exposed areas. Atypical lesions are required to be distinguished from trichoepithelioma, basal cell carcinoma (BCC), molluscum contagiosum, and other sebaceous tumors. Dermoscopy is a non-invasive diagnostic tool that permits the visualization of many morphological features from the skin surface to the mid-dermis (4). Reflectance confocal microscopy (RCM) provides real-time virtual skin biopsies that provide microscopic details of different skin layers up to the papillary dermis (5). Previous reports have demonstrated the dermoscopic manifestations of SGH; however, RCM features of SGH and their correlations with dermoscopic manifestations remain lacking. Therefore, this study aimed to describe the dermoscopic and RCM features of SGH and investigate their relationship.

## Materials and methods

### Study design

This study was conducted at the Department of Dermatology, Chengdu Second People's Hospital, and Dermatology Hospital of Southern Medical University, Chengdu, China, after obtaining institutional review board approval. Informed consent was obtained from all the patients included in this study. Two experienced dermatologists diagnosed patients with SGH based on typical clinical manifestations between March 2018 and January 2022, and histopathological examinations were performed according to the cosmetic or diagnostic accuracy wishes of the patients. The inclusion criterion for our study was lesions located on the face, and only one lesion in each patient was selected randomly for dermoscopy and RCM examination. The exclusion criterion was poor dermoscopic or RCM image quality. The medical records of the enrolled patients were reviewed to analyze their demographic information, duration, lesion size, dermoscopic features, and RCM features.

### Dermoscopic and RCM analysis

Dermoscopic images were recorded using digital video microscopes (Dino-Lite AM7515MZTL, JEDA, Nanjing, China; New Vision UHD 4 K, HONSKIN, Beijing, China). Images were collected in a non-contact polarized mode at 30- and 35-fold magnifications. The RCM images were obtained using a Vivascope 1500 reflectance confocal microscope (Lucid Inc. Rochester, NY, USA) at a standard, horizontal 500 × 500 μm section of the skin at a selected depth from the epidermal surface to the papillary dermis.

During dermoscopy, background color, vascular morphology, scale, and other morphological characteristics of the lesions were assessed, and features such as the architecture of the epidermis, the structure of hair follicles, the morphology of nests in the dermis, and the characteristics of vessels were evaluated. Two authors (Lang Rao and Er-Yi Lin), who were blinded to the histopathological diagnoses, reviewed the dermoscopic and RCM images.

### Statistical analysis

Data were collected and analyzed using SPSS software (version 17.0; SPSS Inc., Chicago, IL, U.S.A.). Descriptive statistics are presented as results of the mean with standard deviations for numeric variables and numerical amounts and percentages for categorical variables. Statistical analysis was performed using Fisher's exact test to evaluate the statistical significance between the two groups, and the results were considered statistically significant at a *P*-value of < 0.05.

## Results

### Clinical characteristics

**Figure 1:**
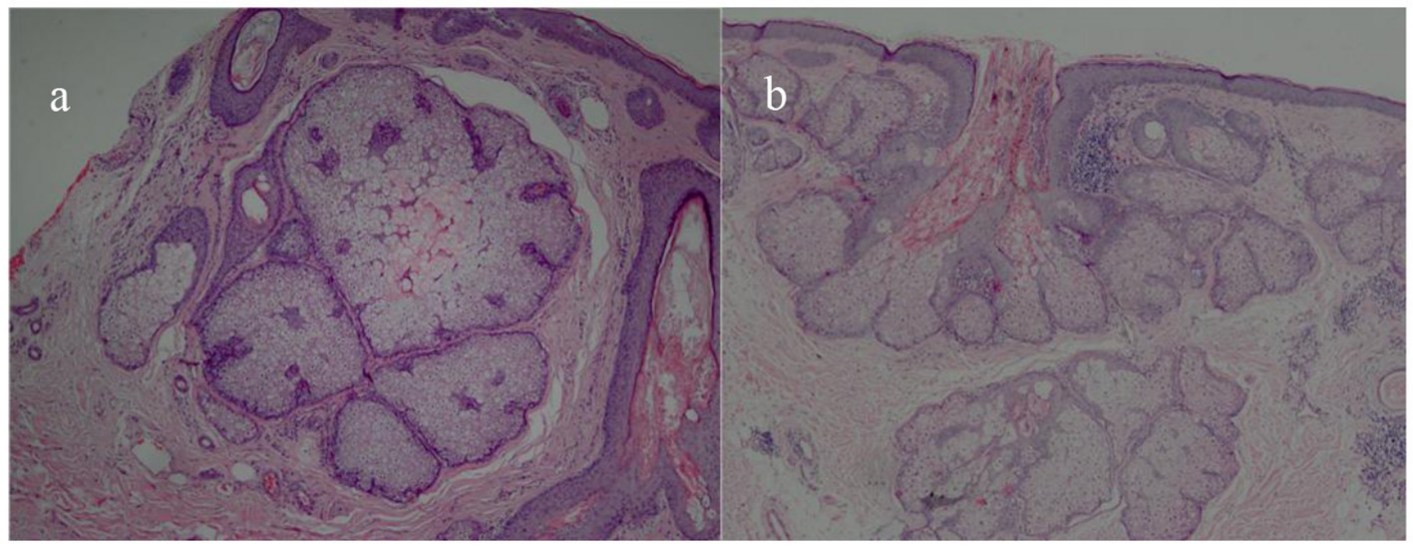
Histopathological pictures of SGH. Multiple hyperplastic sebaceous glands lobules in the dermis (HE × 40) **(a)**; marked hyperplasia with well-differentiated lobules and a central dilated sebaceous gland duct were observed in the upper dermis (HE × 40) **(b)**.

A total of 31 patients, 26 men (83.87%) and 5 women (16.13%), with histologically diagnosed SGH were included in our study, with a mean age of 54.23 ± 12.85 years (23–75 years). The average duration was 21.77 ± 15.35 months (4–72 months). All the lesions were located on the face, and the mean size measured by dermoscopy was 6.18 ± 1.62 mm (3.6–10 mm; Table 1). Among the 31 cases, four patients underwent pathological examination, and the results were all confirmed as SGH (Figure 1).

**Table 1:** Clinical characteristics of the patients.

**Sex**	***n* (%)**
Female	5 (16.13)
Male	26 (83.87)
**Age (years)**	**Mean (range)**
Female	51 ± 16.99 (23–68)
Male	54.85 ± 12.23 (30–75)
**Duration (months)**	
Female	20.6 ± 8.99 (13–36)
Male	22 ± 16.42 (4–72)
**Size (mm)**	
Female	6.92 ± 2.40 (4.3–10)
Male	6.03 ± 1.45 (3.6–8.3)

### Dermoscopic and RCM features of SGH

**Figure 2:**
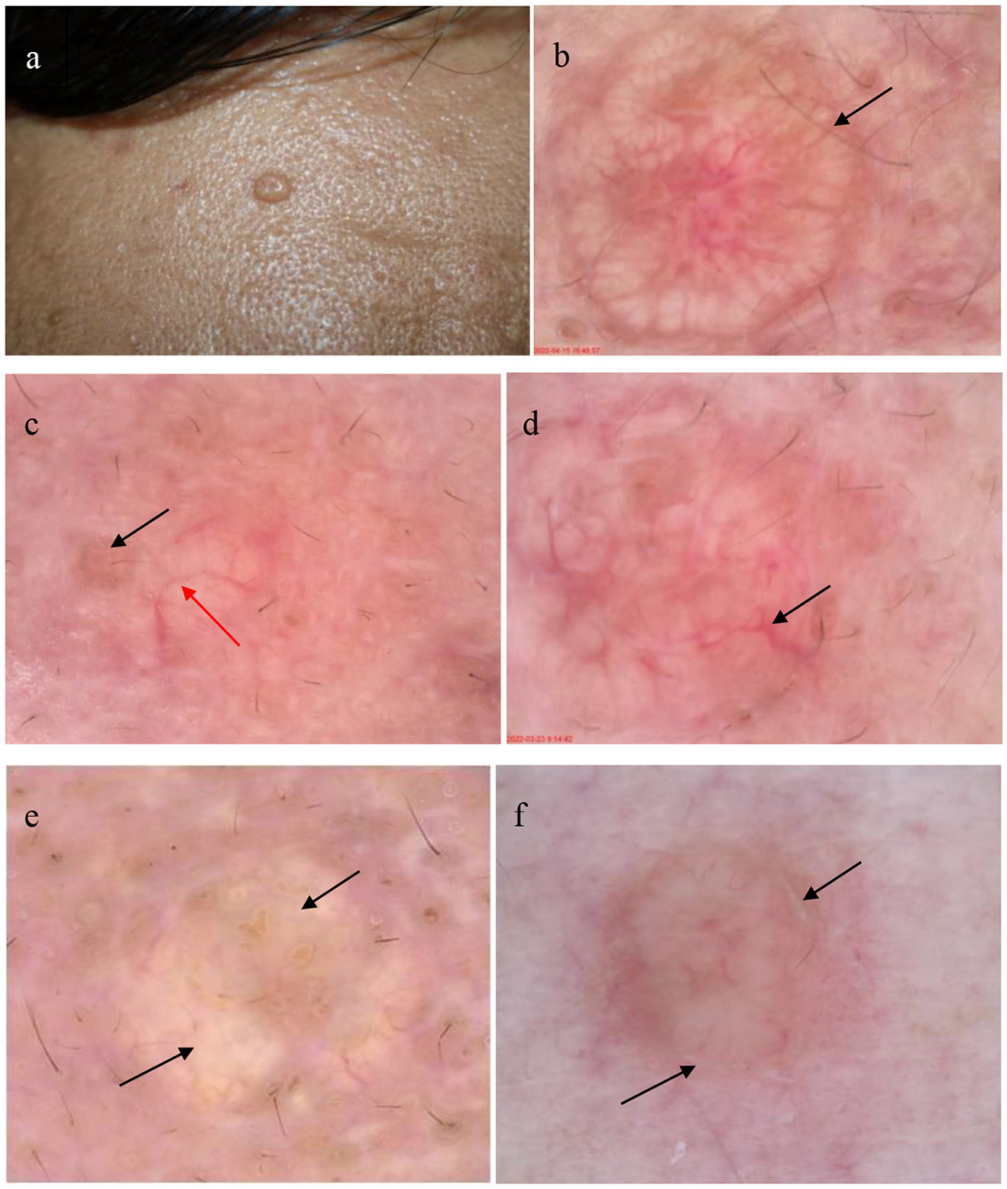
Patient with sebaceous gland hyperplasia (SGH) on the face **(a)**; dermoscopic examination of SGH shows white-yellowish lobulated structures surrounded by crown vessel (black arrow) in the yellowish red background **(b)** (30 × ); dermoscopic image shows typical umbilication (black arrow) in the center with an irregular linear vessel (red arrow) **(c)** (30 × ); dermoscopic image shows the arborizing vessel (black arrow) **(d)** (35 × ); dermoscopic image shows white-yellowish lobulated structures (black arrow) in a faint-yellow background **(e)** (35 × ); and the dermoscopic image reveals white scales (black arrow) around the white-yellowish structures **(f)** (30 × ).

Table 2 and Figure 2 present the specific dermoscopic features of SGH. The most common dermoscopic finding was an aggregated white-yellowish lobulated structure in the center of the lesion (present in 100% of cases). Dermoscopy revealed a yellowish-red and faint-yellow background in 25 cases (80.65%) and six cases (19.35%), respectively. Scales were observed in only three cases (9.68%). Typical umbilication in the center of these white-yellowish structures was also common (61.29%) in patients with SGH. Crown vessels at the periphery of the lesions were observed in 11 patients (35.48%), whereas irregular linear vessels were observed on the surface of the lesions in 18 patients (58.06%).

**Table 2:** Dermoscopic and RCM characteristics of SGH.

	***n* (%)**
**Dermoscopic feature**	
Faint-yellow background	6 (19.35)
Yellowish-red background	25 (80.65)
White-yellowish lobulated structure	31 (100)
Umbilication	19 (61.29)
Crown vessel	11 (35.48)
Irregular linear vessel	18 (58.06)
Arborizing vessel	2 (6.45)
Scale	3 (9.68)
**RCM feature in the epidermis and superficial dermis**	
Honeycombed pattern	31 (100)
Streaming in epidermis	4 (12.90)
Dilated follicular infundibulum	15 (48.39)
Morulae-shaped sebaceous lobules	31 (100)
Dilated vessels	26 (83.87)
Inflammatory cells in the superficial dermis	2 (6.45)
Sebaceous lobules surrounded by hyper-refractile collagen fibers	13 (41.94)

RCM, reflectance confocal microscopy; SGH, sebaceous gland hyperplasia.

**Figure 3:**
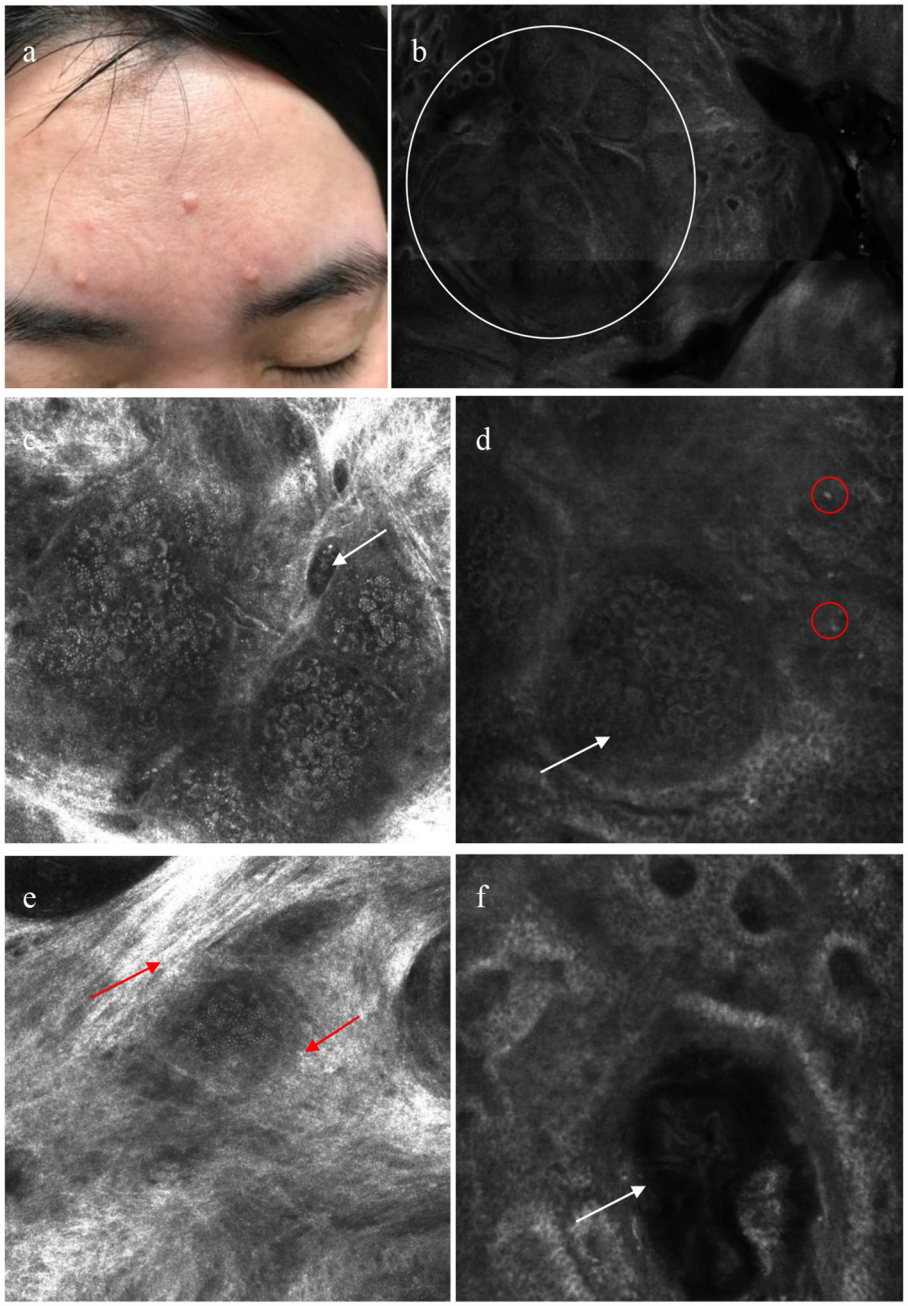
Patient with sebaceous gland hyperplasia (SGH) on the face **(a)**; reflectance confocal microscopy (RCM) shows aggregated morulae-shaped sebaceous lobules (white circle) **(b)**; dilated vessel (white arrow) **(c)**; RCM images reveals morulae-shaped sebaceous lobules (white arrow) with a few inflammatory cells (red circle) in superficial dermis **(d)**; sebaceous lobules surrounded by hyper-refractile cytoplasm (red arrow) **(e)**; a dilated follicular infundibulum with medium and high refraction materials contained (white arrow) **(f)**.

Table 2 and Figure 3 show the summary RCM features of the SGH. All lesions showed a honeycomb pattern in the epidermis and typical morula-shaped sebaceous lobules in the dermis. In addition, the sebaceous lobules were composed of round cells with a speckled cytoplasm and dark nuclei surrounded by hyper refractile collagen fibers in 13 (41.94%) cases. A dilated follicular infundibulum was observed in 15 (48.39%) patients, and most lesions (83.87%) displayed dilated vessels in the superficial dermis. However, inflammatory cells were observed in only two patients (6.45%).

## Discussion

SGH is a benign and common condition of the sebaceous glands. The sebaceous glands consist of holocrine acini attached to a common excretory duct and follicle, collectively comprising the pilosebaceous unit. In SGH, the sebaceous glands are normal in structure but increase in number (6). All acini are attached to the central duct, which may become dilated, and the follicular infundibulum when the sebaceous glands increase. In our study, the central umbilication detected using dermoscopy in 19 cases (61.29%) correlated with a dilated central follicular infundibulum containing medium- and high-refractive structures present in 15 cases (48.39%) determined via RCM examination. Shahriari et al. (7) have reported that the dilated central duct and follicular infundibulum contain sebum and keratin debris. Therefore, the features of umbilication in SGH observed by dermoscopy were different from those of molluscum contagiosum. In the molluscum contagiosum, umbilication corresponds to molluscum bodies, which show round, well-circumscribed lesions with a central round cystic area filled with a brightly refractile material in RCM features (8).

Dermoscopic lesions mostly revealed a yellowish-red background in 25 cases (80.65%) and a faint-yellow background in 6 cases (19.35%). We observed that lesions in female patients were more likely to have a faint-yellow background. Among the 25 cases with a yellowish-red background, 24 cases were male and one case was female, and among the 6 cases with a faint-yellow background, 2 cases were male and four cases were female (*P* < 0.05). This finding may be because Asian women generally have lighter skin tones than men. This study showed that white-yellowish lobulated structures were the most common dermoscopic features of SGH, corresponding to the morulae-shaped sebaceous lobules observed under RCM. Bryden et al. (9) named it a “cumulus sign,” which can be easily distinguished from the blue-gray ovoid nest in BCC under dermoscopy. Moreover, the presence of morula-shaped structures in SGH, compared to the bright tumor islands observed in BCC and trichoepithelioma, indicated its value as a distinguishing feature. The morula-shaped structures have been described in sebaceous nevi, and further identification is required in clinical manifestations and histopathological examinations. Additionally, white-yellowish lobulated structures are warranted to be distinguished from the white circular structures of squamous cell carcinoma (SCC). The disordered arrangement of epidermal cells and speckle-nucleated cells in SCC under RCM may provide a more powerful basis for differential diagnosis (Table 3).

**Table 3:** Main dermatoscopy and RCM findings and their histopathologic correlation.

	**Dermatoscopy**	**RCM**	**Histopathology**
SGH	White-yellowish lobulated structure Umbilication Crown, irregular, arborizing vessels	Morulae-shaped sebaceous lobules Dilated follicular infundibulum Dilated dermal vessels	Hyperplastic sebaceous glands lobules Dilated sebaceous gland duct and the follicular orifice Dilated dermal vessels
BCC	Blue ovoid nests and blue globules/leaf-like structures/spoke wheel areas Gray-dot granules Arborizing blood vessels Porcelain white areas Erosion/ulceration	Bright tumor cords/islands with peripheral palisading or dark silhouettes Dark clefts around tumor islands Plump-bright cells in the dermis Linear tortuous blood vessels in the dermis Bright collagen bundles around the tumor Erosion/ulceration	Islands of basaloid tumor cells Stromal retraction Melanophages/inflammatory cells Dilated dermal vessels Thickened collagen fibers in the dermis Loss of epidermis
SCC	White circles Central keratin/Scale- Linear/polymorphous vessels Erosion/ulceration Pink/White-yellowish structureless areas	Keratin pearl Hyperkeratosis/Parakeratosis Architectural disarrangement Dilated dermal vessels Erosion/ulceration Speckled nucleated/targetoid cells or nest-like structures in the dermis	Keratin pearl Hyperkeratosis/Parakeratosis Disordered arranged epidermis cells Dilated dermal vessels Loss of epidermis Irregular squamous cells
Trichoe-pithelioma	Shiny-white homogenous structures Milia-like keratin cysts Short, thin telangiectasias –	Leaf-like lumps of tumor islands Horn cysts Dilated dermal vessels Brightly refractile stroma around the tumor islands	Clumps of basaloid cells in the dermis Angular cysts Dilated dermal vessels Peripheral fiber hyperplasia
Molluscum contagiosum	Yellowish-white, roundish, or poly-lobular amorphous structure Crown of fine, linear vessels	Hypo-refractive roundish lobules containing hyper refractive cells Dilated dermal vessels	Enlarged keratinocytes containing intracytoplasmic viral inclusions Dilated dermal vessels

Furthermore, irregular linear vessels (58.06%) were the most common vascular structures in SGH, followed by crown vessels (35.48%). This finding differs from that of Argenziano and Oztas (10, 11), who showed that the crown vessels were the most common vascular pattern. Arborizing vessels were observed in two (6.45%) cases, and identification of non-pigmented BCC through other structures or RCM examination is required. Different dermoscopic vascular patterns presenting as dark dilated vessels in the RCM were observed in 26 patients (83.87%). In the dermoscopic analysis in our study, the monomorphic vessel patterns were mainly observed, whereas Cheng et al. (12) reported that the polymorphic vessel pattern often indicates a malignant tumor, such as sebaceous carcinoma.

In 13 cases (41.94%), the sebaceous lobules surrounded by hyper refractile reticulated collagen fibers were observed, which is also supported by the findings of Fraga-Braghiroli et al. (13). Notably, we observed that the reticulated collagen fibers surrounding the sebaceous lobules were more likely present in patients younger than 50 years (*P* < 0.05), possibly due to the loss of collagen fibers and the relaxation of reticular fibers with increasing age.

In summary, we examined SGH using dermoscopy and RCM and compared them with the corresponding histopathological findings. We observed that there are three main dermoscopic and RCM features noticeable in SGH, which are helpful for diagnosis and differential diagnosis, as follows: (i) white-yellowish lobulated structures in a faint-yellow or yellowish-red background, corresponding to the morulae-shaped sebaceous lobules in RCM; (ii) umbilication in the center of the lesion, corresponding to dilated follicular infundibulum in RCM; and (iii) crown or linear-irregular vessels, corresponding to dark dilated vessels in RCM. The main limitation of our study was the relatively small number of patients. Additionally, we only selected lesions on the face, and no controls were included. Further studies with large sample sizes are needed to test our findings and explore the usefulness of dermoscopy and RCM as non-invasive diagnostic tools for SGH.

## Data availability statement

The original contributions presented in the study are included in the article/supplementary material, further inquiries can be directed to the corresponding author.

## Ethics statement

Written informed consent was obtained from the individual(s), and minor(s)' legal guardian/next of kin, for the publication of any potentially identifiable images or data included in this article.

## Author contributions

LR contributed to imaging analysis, figure editing, and critical review of the manuscript. E-YL contributed to the pathological analysis, figure editing, and critical manuscript review. W-JW supervised clinical studies and critically reviewed the manuscript. Y-FC conceived this concept. All authors contributed to the article and approved the submitted version.
